# Early and extensive CD55 loss from red blood cells supports a causal role in malarial anaemia

**DOI:** 10.1186/1475-2875-10-386

**Published:** 2011-12-29

**Authors:** Moses Gwamaka, Michal Fried, Gonzalo Domingo, Patrick E Duffy

**Affiliations:** 1 Mother-Offspring Malaria Studies Project, Muheza Designated District Hospital, Muheza, Tanzania; 2 Sokoine University of Agriculture, Morogoro, Tanzania; 3 Biomedical and Environmental Thematic Group, Ifakara Health Institute (IHI), PO Box 53, Ifakara, Tanzania; 4 Seattle Biomedical Research Institute, Seattle, WA, USA; 5 Laboratory of Malaria Immunology and Vaccinology, National Institute of Allergy and Infectious Diseases, NIH, Rockville, MD 20892, USA; 6 Programs for Appropriate Technologies in Health (PATH), Seattle, WA 98121, USA

## Abstract

**Background:**

Levels of complement regulatory proteins (CrP) on the surface of red blood cells (RBC) decrease during severe malarial anaemia and as part of cell ageing process. It remains unclear whether CrP changes seen during malaria contribute to the development of anaemia, or result from an altered RBC age distribution due to suppressive effects of malaria on erythropoiesis.

**Methods:**

A cross sectional study was conducted in the north-east coast of Tanzania to investigate whether the changes in glycosylphosphatidylinositol (GPI)-anchored complement regulatory proteins (CD55 and CD59) contributes to malaria anaemia. Blood samples were collected from a cohort of children under intensive surveillance for *Plasmodium falciparum *parasitaemia and illness. Levels of CD55 and CD59 were measured by flow cytometer and compared between anaemic (8.08 g/dl) and non- anaemic children (11.42 g/dl).

**Results:**

Levels of CD55 and CD59 decreased with increased RBC age. CD55 levels were lower in anaemic children and the difference was seen in RBC of all ages. Levels of CD59 were lower in anaemic children, but these differences were not significant. CD55, but not CD59, levels correlated positively with the level of haemoglobin in anaemic children.

**Conclusion:**

The extent of CD55 loss from RBC of all ages early in the course of malarial anaemia and the correlation of CD55 with haemoglobin levels support the hypothesis that CD55 may play a causal role in this disorder.

## Background

Anaemia is a common devastating complication of malaria caused by *Plasmodium falciparum*. The pathogenesis of malarial anaemia is complex, multifactorial and incompletely understood. Direct destruction of infected red blood cells (RBC) during malaria seems to be a relatively minor contributing mechanism because parasite densities do not correspond to the severity of anaemia [1,2]. As a result, malarial anaemia is considered to arise mainly from defective erythropoiesis or from removal of uninfected RBC [1].

Unusually low numbers of reticulocytes in the peripheral blood indicate ineffective erythropoiesis and are frequent in chronic malaria cases [3]. During acute malaria, anaemia is thought to arise mainly from the loss of uninfected RBC [1,2] although the mechanism for this is not completely understood. Uninfected RBC may be prematurely removed from the circulation by either phagocytic immune cells or complement attack. In Kenya, children with severe malarial anaemia demonstrated increased erythrophagocytosis as well as decreased levels of complement regulatory proteins (CrP) including complement receptor 1 (CR1) and decay accelerating factor (CD55) [4]. Decreases in CrP levels may predispose RBC to destruction by complement activation, thus contributing to the development of anaemia. CrPs are absolutely required to protect RBC from spontaneous complement damage [5] and CrP deficiencies render RBC more susceptible to complement damage [6,7].

Although levels of other surface molecules are known to vary as RBC age [8-11], changes in the CD55 and CD59 levels in relation to cell aging during malaria have not been examined. Impaired RBC production and shortened RBC survival occur during malaria, and together have the effect of reducing the production of new RBC and abbreviating the life span of old RBC [3]. Thus, altered RBC age distributions might contribute to apparent changes in CrP levels during malarial anaemia. This study compared surface glycosylphosphatidylinositol (GPI)-anchored complement regulatory proteins levels on RBC of different ages in infected children with or without recent onset of malarial anaemia.

## Methods

### Study population and clinical procedures

Demographic and laboratory characteristics of the study population are presented in Table 1.

**Table 1:** Demographic characteristics of study groups

	Healthy control	Non anaemic	Anaemic children	P Value
Cases (Male/Female)	N = 21 (12/9)	N = 34 (16/18)	N = 50 (32/18)	

Haemoglobin g/dl (SD)	11.64 (1.16)	11.42 (1.68)	8.08 (1.51)	< 0.0001*

Age (Months)	18.95 (11.52)	19.45 (8.4)	17.62 (6.1)	0.0936

Parasite density/200wbc)	-	2884.2(3122.5)	2700.64 (3458)	0.9715

Haemoglobin level are presented as means (Standard deviation) Data on age and parasite density are presented in Median (Interquartile range) values* Significant difference between anaemic and non anaemic children

The study population included children participating in a longitudinal birth cohort study managed by the Mother Offspring Malaria Studies (MOMS) Project. The study was based at Muheza Designated District Hospital (DDH), near the north-eastern coast of Tanzania, an area of intense malaria transmission.

Children participating in the birth cohort were examined by an assistant medical officer every two weeks during infancy and every month thereafter; each examination included blood smear analysis. Venous blood samples were collected at 3, 6 and 12 months of age, then once after every six months in the second and third years of life and during any episode of parasitaemia or illness. Children were treated for any malaria episode, and efficacy of treatment was confirmed one week later by repeat blood smear. Capillary blood was drawn into heparinized tubes for preparation of blood smears, and venous blood for blood cell counts was drawn into a syringe containing the anticoagulant Citrate/Phosphate/Dextrose. Parasites were detected by microscopic examination of Giemsa-stained thick and thin smears prepared from capillary or venous blood. Parasitaemia was quantified as the number of parasitized RBC per 200 white blood cells in the thick smear, and parasite species were determined by examination of the thin smear. Haemoglobin levels and other haematological parameters were determined by haematology analyzer Cell Dyne^® ^1200 (Abbott Diagnostics Division, IL, USA).

The analyses for this study involved 105 children aged between six and 30 months. Of these, 84 children were sequentially enrolled after being diagnosed as having malaria and 21 healthy children served as controls. Healthy control children were enrolled after being proved to have normal haemoglobin levels, free from *P. falciparum *infection both symptomatically and microscopic blood smear examination, and had no record of parasitaemia for at least three months prior to sampling. Clinical malaria was defined as asexual *P. falciparum *parasitaemia by blood smear coupled with symptoms suggestive of malaria, most commonly fever ≥ 37.5°C. Anaemia was defined as haemoglobin concentration below 10 g/dL. Children with sickle cell anaemia (HbSS), Glucose 6 phosphate dehydrogenase enzyme deficiency (both hemizygous males and homozygous female) and 3.7 kb deletion alpha-thalassemia (heterozygous and homozygous) were excluded from this analysis.

### Ethical issues

Women presenting at Muheza DDH for delivery were invited to enroll their offspring in the cohort, and provided signed consent prior to the participation of their newborns in the birth cohort study. Protocols for procedures used in this study were approved by the International Clinical Studies Review Committee of the Division of Microbiology and Infectious Diseases at the US National Institutes of Health, and ethical clearance was obtained from the Institutional Review Boards of Seattle Biomedical Research Institute and the National Institute for Medical Research in Tanzania.

### Determination of RBC age profiles

RBC were separated into age subpopulations by a density gradient centrifugation method as described previously [12,13] with minor modifications. RBC were separated from plasma and leukocytes by centrifugation at 600 × *g *for 5 minutes. After removing the plasma and leukocytes, RBC were washed in PBS buffer twice, and a 100 μl blood pellet was resuspended in RPMI 1640. Percoll (Sigma-Aldrich, St. Louis, MOUSA) density gradients were used to separate RBC into fractions of varying ages. Percoll/5% sorbitol (W/V) was prepared by dissolving 5 g of sorbitol in 10 ml RPMI 1640 then mixing with 90 ml of Percoll. This solution was diluted with RPMI 1640 to make 90%, 80%, 70%, 60% and 40% Percoll solutions, corresponding to the following densities 1.099 g/ml, 1.082 g/ml, 1.070 g/ml, 1.061 g/ml and 1.046 g/ml respectively. The gradients were prepared in 15 ml plastic tubes (1 ml of each fraction), with the highest Percoll concentration at the bottom. Thereafter, 0.1 ml packed RBC was diluted in 5 parts of RPMI 1640 then layered on top of the Percoll gradient, and centrifuged at 1075 × *g *for 20 minutes at room temperature. Each fraction of cells was aspirated and transferred to a new tube, washed twice in RPMI 1640, then suspended and pelleted by centrifugation at 600 × *g *for 5 minutes. The supernatant was removed, and the pelleted cells resuspended by adding 200 μl of RPMI 1640. The number of red cells in each fraction was counted by haematology analyzer, and the cells in each age group were calculated as proportions of the total number of cells.

### Measurement of CD55 and CD59

Red blood cells were washed twice in PBS buffer supplemented with 1% BSA and 0.1% NaN_3 _then suspended in the same buffer at 1 × 10^6 ^cells/ml. To label CrP, the RBC were incubated with monoclonal antibodies against human CD55 and CD59, according to manufactures instructions (BD Biosciences-PharMingen, USA) conjugated to Cyanine 5 (CY5) and Phycoerythrin (PE), respectively. Irrelevant monoclonal antibodies of the same isotype were used as negative controls (Molecular Probes, USA). The samples were incubated at 4 C in the dark for 30 minutes. The RBC were washed twice in buffer, resuspended in PBS and analysed immediately by flow cytometer (Flomax, Partec, Germany). The cells were excited with 488 nm argon ion laser, and the logarithmic orange (PE) and red (CY 5) fluorescences were measured through FL2 and FL3 detectors respectively. RBC were gated on the basis of their logarithmic amplification of the light scatter properties. One thousand events were acquired in replicate for each sample. The results were presented as mean fluorescence intensity (MFI) of RBC with specific immunofluroescence above the background fluorescence as determined by isotype controls [4].

### Detection of phosphatidylserine exposure

Annexin V-FITC apoptosis detection kit (Sigma-Aldrich) was used for the detection of phosphatidylserine on RBC surfaces. The cells were washed twice in PBS and then resuspend in 1 × binding buffer (100 mM HEPES/NaOH, pH 7.5 containing 1.4 M NaCl and 25 mM CaCl2) at a concentration of approximately1 × 10^6 ^cells/ml. Then 1 μl of Annexin V-FITC (Sigma) was added in each 100 μl of cell suspension and mixed thoroughly by gently vortex. The tubes were incubated at room temperature for exactly 10 minutes in the dark. Thereafter, 400 ml of binding buffer was added to each tube. Analysis by flow cytometer (Flomax - Partec^® ^- Germany) was performed within 1 hr. The negative control was prepared each time by following all steps for PS staining except that the Annexin V-FITC addition step was skipped.

### Validation of the Percoll density gradient method

Percoll density gradient method was validated by assessing the relationship between RBC density and age related modifications. Blood for this purpose was obtained from seven healthy donors, and then processed similar to other study subjects. After density gradient separation, each RBC fraction was analysed for the mean corpuscular volume (MCV), level of phosphatidylserine exposure to surface membranes and levels of CD55 and CD59.

### Data analysis

Statistical analysis was performed using StatView version 5.0.1 (SAS Institute Inc. Cary, NC, USA) software package. The Mann-Whitney test was used to test differences between groups and Spearman rank correlation to examine relationships between variables. Multivariate linear regression analyses were used to determine the relationship between parasite density, CrP levels and child's age. A *P *value ≤ 0.05 was considered statistically significant.

## Results

### Study population and characteristics

Demographic and laboratory characteristics of the study population are presented in Table 1.

### RBC Age profiles

In most samples the cells separated into four distinct bands following centrifugation on Percoll density gradients and were designated as young for top band (60% Percoll solution), mature for second band from top (70% Percoll solution), old for third band form top (80% Percoll solution), and very old for the bottom band (90% Percoll solution) subsets [10]. An additional layer containing mostly leukocytes, infected RBCs and few reticulocytes as assessed by microscopy was observed at the 40% Percoll solution layer in few samples, but this heterogeneous pool was not included in the current analysis.

Analysis of blood obtained from healthy subjects to validate the Percoll density method showed that MCV measurements decreased as cells increased in density, the difference between young cells and oldest fraction was statistically significant (Table 2). Similarly, the mean fluorescence intensities for CD55 and CD59 decreased with increasing density. Annexin V binding was higher in the bottom band (older RBC subsets) than in the younger populations (Table 2), suggesting that the exposure of endogenous phosphatidyiserine on RBC surface increased with cell age and is concentrated in the oldest fraction as reported previously [10].

**Table 2:** Parameters used to validate the age-density relationship in RBC fractions separated by Percoll density gradient method

Erythrocyte Age Fraction	Density g/ml	% of cells	MCV			PS- MFI			CD55- MFI			CD59- MFI		
			
			Mean ± SD	% change	P	Mean ± SD	% change	P	Mean ± SD	% change	P	Mean ± SD	% change	P
Young	1.061	23.5 ± 26.64	73.86 ± 1.29	0	NA	1.36 ± 0.182	0	NA	2.55 ± 0.512	0	NA	3.39 ± 0.775	0	NA

Mature	1.07	44.37 ± 18.53	73.43 ± 1.39	0.58	0.1	1.43 ± 0.088	5.15	0.1416	2.11 ± 0.568	17.25	0.139	3.18 ± 0.692	6.19	0.035

Old	1.082	25.86 ± 15.99	73.00 ± 1.41	1.64	0.08	1.56 ± 0.177	14.71	0.1312	1.94 ± 0.617	23.92	0.114	3.02 ± 0.564	10.91	0.023

Very old	1.099	6.41 ± 7.94	72.29 ± 1.6	2.13	0.01	1.67 ± 0.23	22.79	0.0027	1.89 ± 0.408	25.88	0.008	2.59 ± 0.475	23.59	0.0017

MCV - Mean corpuscular volume, PS- Phosphatidylserine, MFI = Mean Fluorescence Intensity

**Figure 1:**
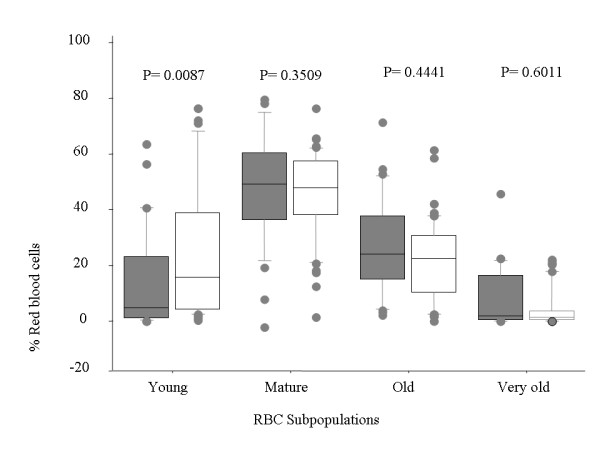
**Levels of young RBC increase during new onset malarial anaemia**. RBC of different ages (subsets) presented as a proportion of total RBC count. Blood was obtained from children with malarial anaemia (open boxes) and from non-anaemic infected children (shaded boxes), and compared for differences in proportions of RBC at different ages. Each box represents the interquartile range (25-75%) of values, the whiskers represent 10% and 90% values, the middle line represents median value and the circles represent patients who fell outside the 10% and 90% range.

Overall analysis of RBC age profile from malaria infected children indicated that the mean proportional of cells in the top band was 20.82% xs(± 19.3), in the second band was 50.56% (± 17.69) in the third band was 23.4% (± 15.72) and 5.62% (± 4.52) in the bottom band. Compared to children in other groups, blood obtained from anaemic donors displayed a significant increase in the proportion of young RBC, and non-significant changes in other subpopulations (Figure 1). Neither the child's age nor the parasite density correlated with RBC age profile (Spearman rank test, *P *> 0.05 for all comparisons in both anaemic and non-anaemic groups).

### Cytofluorometry

Overall levels of CD55 but not CD59 were higher in the healthy controls than in other groups but this difference was only significant for the anaemic malaria cases. CD55 MFI levels were significantly higher in the healthy control group in all RBC subsets when compared to anaemic malaria cases i.e. median (IQR = 25-75%) for young [2.52 (0.36) versus 2.15(0.8), *P *= 0.0074]; mature [2.03 (0.46) versus 1.83 (0.44), *P *= 0.0026]; old [1.82 (0.47) versus 1.65 (0.33), *P *= 0.0327] and very old [1.67 (0.38) versus 1.69 (0.38), *P *= 0.0399] but not to the non-anaemic malaria cases i.e. median (IQR = 25-75%) for young [2.52 (0.36) versus 2.28(0.94), P = 0.2592]; mature [2.03 (0.46) versus 2.1(0.68), *P *= 0.4356]; old [1.82 (0.47) versus 1.87(0.34), *P *= 0.9791] and very old [1.67 (0.38) versus 1.75(0.29), *P *= 0.3080]. CD59 levels were low during malaria compared to normal controls but the differences were not significant for all RBC subsets.

**Figure 2:**
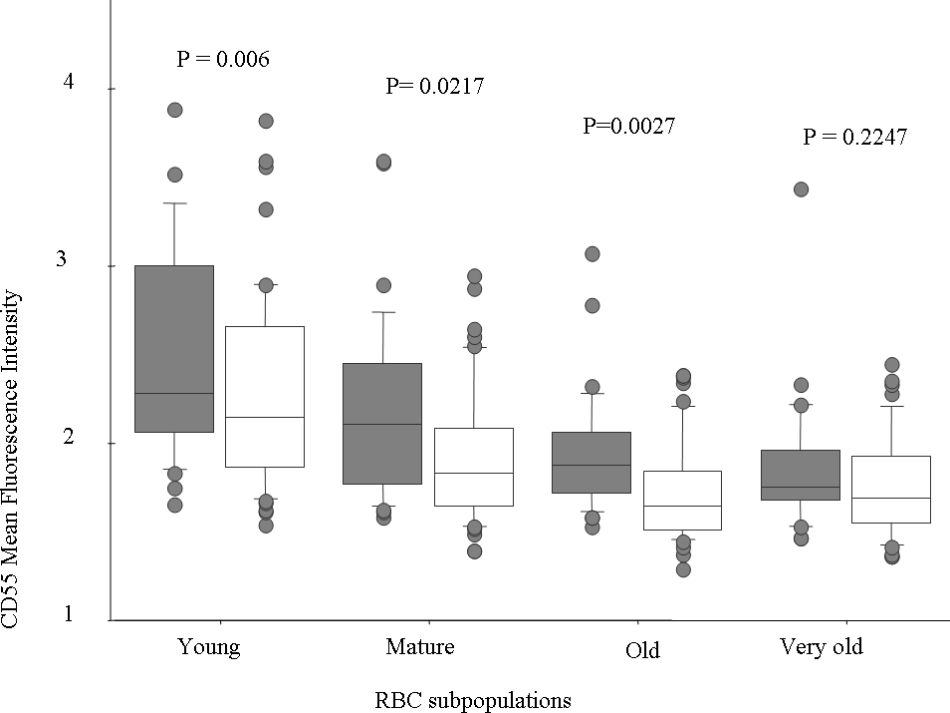
**CD55 levels decrease on RBC of all ages during new onset malarial anaemia.** Levels of CD55 on the surface of RBC obtained from children infected with P. falciparum with (open boxes) or without (shaded boxes) anaemia. Each box represents the interquartile range (25 -75%) of values, the whiskers represent 10% and 90% values, the middle line represents median value and the circles represent patients who fell outside the 10% and 90% range. CD55 levels differed significantly on the basis of RBC age and anaemia status. P value indicates significance of relationship to anaemia status.

**Figure 3:**
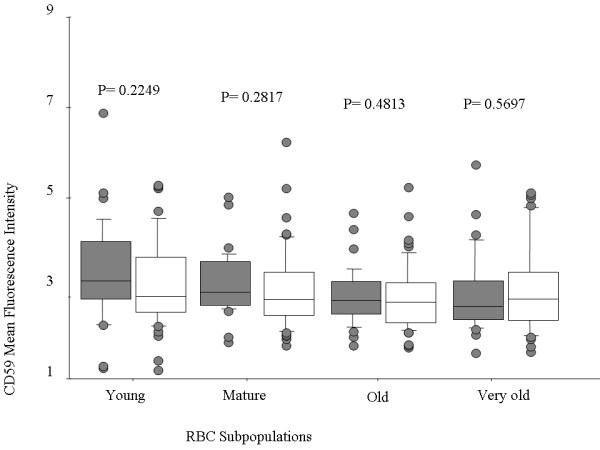
**CD59 levels do not change significantly on RBC of all ages during new onset malarial anaemia. ** Levels of CD59 on the surface of RBC obtained from children infected with P. falciparum with (open boxes) or without (shaded boxes) anaemia. Each box represents the interquartile range (25 -75%) of values, the whiskers represent 10% and 90% values, the middle line represents median value and the circles represent patients who fell outside the 10% and 90% range. CD59 levels varied significantly on the basis of RBC age but not anaemia status. P value indicates significance of relationship to anaemia status.

Levels of CD55 and CD59 decreased progressively as RBC aged, in all children. CD55 levels were lower in anaemic compared to non-anaemic children for all RBC age subsets, and these differences were significant for all except the very old RBC subset (Figure 2). CD59 did not differ significantly between anaemic and non-anaemic children in any RBC subset (Figure 3).

CD55 levels correlated significantly with the level of haemoglobin in anaemic (Spearman rank test: young RBC, *r *= 0.506, *P *= 0.001; mature RBC, *r *= 0.421, *P *= 0.0063; old RBC, *r *= 0.526, *P *= 0.0008; very old RBC, *r *= 0.375, *P *= 0.0246) but not in non-anaemic children with malaria. CD59 level did not correlate with haemoglobin in either group of children.

CrP levels did not vary with child's age. CD55 but not CD59 levels were negatively associated with parasite density. The relationship of CD55 to parasite density was significant for both anaemic (*r *= -0.386; *P *= 0.0069) and non-anaemic populations (*r *= -0.469; *P *= 0.0063). Multivariate linear regression analyses for parasite density, CD55 levels and child's age as independent variables, and haemoglobin level as the dependent variable indicated that decreases in CD55 was associated with the odds (OR 95% CI) for low haemoglobin levels of 0.19 (0.05-0.83).

## Discussion

The results confirm previous findings that CD55 levels on the surface of RBC are lower in children with malarial anaemia [4], and in addition indicate that CD55 loss begins early in the course of disease and affects RBC of all ages. It is also shown that CD55 and CD59 levels decrease progressively as RBC age. Altered RBC age profiles during *P. falciparum *infections could therefore modify complement regulatory proteins (CrP) levels, but specifically do not account for the decrease in CrP during early malarial anaemia. The correlation between CD55 and haemoglobin levels in anaemic children suggests that CD55 loss may at least partially mediate the disease.

This study focused on acute episodes of mild to moderate anaemia. Children in the cohort were monitored intensively with routine blood smears, clinical examinations and prompt treatment in case of illness, and therefore chronic malaria cases were unlikely. Significant increase in number of young RBC in anaemic children suggests body's attempt to correct the reduction in cell mass resulting from malaria infection, and also suggests that these cases were acute and not chronic episodes [14]. Earlier studies focused on hospitalized children with severe malarial anaemia and presumably many of these were chronic cases [4]. Although CD55 levels were found to be higher in young RBC, the increase in the fraction of young RBC during malarial anaemia in this cohort did not lead to an overall increase of CD55 levels. RBC of all ages had lower CD55 levels in anaemic versus non-anaemic children, suggesting that an active process may be removing CD55 from RBC in cases of malarial anaemia.

Red blood cells were separated into fractions of different ages using Percoll density gradients method. Density gradient separation is a well validated technique which yields distinct cell populations with progressive shift in the cell age with density, and differ by physical and biochemical properties [15-17]. Decrease in the mean corpuscular volume, expression of GPI-anchored glycoproteins (CD55 and CD59) and increased exposure of phosphatidylserine (PS) on the external leaflet of cell membranes are related to the RBC aging process [10-18]. Indeed, in normal donor blood samples, there was a decrease in MCV, CD55 and CD59 inversely related to RBCs density. These observations, together with the increased exposure of phosphatidylserine (PS) in the more dense RBCs, supports the notion that in density -separated subsets, an enrichment of young, mature, old and very old RBCs subsets was attained.

Apparently, this is the first study to show variations in CD55 and CD59 levels in relation to RBC age during malaria. Earlier study reported a progressive decrease in CD55 and CD59 levels with RBC age in healthy donors [18,19]. Reductions in CD55 and CD59 molecules during RBC aging in healthy individuals suggest that non-pathological mechanisms exist to mediate CrP loss throughout RBC life. These mechanisms are not fully understood, although several studies suggest that CrP molecules are lost from RBC through vesicle formation and extrusion from the cell surface [11-20]. Additional mechanisms may include proteolytic cleavage of CrP during transport and clearance of immune complexes from the RBC surface in the liver and spleen [8]. However, the loss of RBC surface molecules during malarial anaemia appears to be a selective process. CD55 but not CD59 levels were significantly lower in anaemic donors. It is suggested that an active process separate from physiological cell ageing may be taking place during malaria that preferentially removes specific RBC surface molecules.

Alternatively the same mechanism responsible for physiological loss may be taking place, but at an accelerated rate for CD55. A process resembling transfer reaction of CR1 has been proposed to explain the loss of CD55 on RBC during malaria [21]. During transfer reaction immune complexes are removed from RBC surfaces by phagocytes. In healthy individuals, RBC act as passive shuttles for the transport of complement-coated immune complexes from the circulation to the reticuloendothelial phagocytes in the liver and spleen.

The correlation between CD55 and haemoglobin as observed in this study suggests that CD55 depletion contribute to RBC loss during malarial anaemia. Erythrophagocytosis resulting from C3b deposition on RBC could explain the concomitant decrease of CD55 and haemoglobin. Severe malarial anaemia is associated with elevated levels of circulating immune complexes [22,23], which could be adsorbed by CD55 and subsequently transferred to macrophages [21]. The loss of CD55 during this process could compromise its regulatory function and allow the deposition of opsonin C3b on RBC [24,25], leading to increased RBC destruction by the phagocytes in the reticuloendothelial tissues. Complement binding to RBC has been reported to be associated with macrophage activation and reduced haemoglobin in *P. falciparum *malaria [26].

Alternatively, direct lysis of RBC by membrane attack complex (MAC) could explain the concomitant decrease of CD55 and haemoglobin, but seems less likely. Children with severe malarial anaemia have been reported to have low levels of CR1 and CD55 [3]. Such deficiencies would likely render the RBC vulnerable to complement attack, especially during complement activation which is common during malaria [27]. However, CD59 did not decrease significantly during anaemia episodes in this or earlier studies [4], which imply that complement-mediated lysis is not the likely mechanism for RBC loss during malaria. CD59 is a principal regulatory protein of complement attack [17]. Weisner *et al *[28] found that despite the activation of all lytic complement factors, no complement-mediated lysis of RBC occurred in the presence of functional intrinsic CD59. Because CD59 levels do not change significantly during malarial anaemia, this probably limits complement-mediated lysis.

The differential loss of CD55 and CD59 may be related to their different roles in protection from complement attack. Activation of complement occurs in a step-wise fashion, and each regulatory protein acts at a different step in the cascade. CD55 acts at the initial enzymatic step to prevent the activation of C3 to C3b by accelerating the dissociation of the C3 convertase C4-2a and C3bBb [28-30]. CD59 prevents formation of polymeric C9 complex at the final step of MAC assembly [31]. At least during acute malarial anaemia, CR1 and CD55 may sufficiently regulate the complement cascade to limit formation of MAC, thereby consuming CD55 and sparing CD59.

Demonstration of higher amounts of C3 activation and degradation fragments bound to the RBCs of the anaemic children and an inverse correlation with CD55 would be another approach to support the hypothesis that CD55 loss supports the causal role in malarial anaemia. Moreover, demonstration of C3b on the CD55 low cells, would argue that sublytic C5b-9 induced the ectocytosis of infected and bystander cells, which resulted in the preferential loss of CD55 over CD59. This phenomenon was not explored in the current study, but earlier studies by other researchers did demonstrate an increase in C3b deposition on low red cell CR1 and CD55 levels in children with severe malarial anaemia [24,25].

## Conclusions

Reductions in CD55 during malarial anaemia are evident in RBC of all ages, correlate with haemoglobin level, and develop early in the course of disease. Although the RBC age distribution changes during malarial anaemia, and CD55 levels decrease as RBC age, these changes do not account for the CD55 loss seen in children with new onset disease. Taken together, the results support the hypothesis that CD55 loss may play a causal role in malarial anaemia. It is suggested that the loss of CD55 during malaria compromises the complement regulatory function thereby allows the deposition of opsonin C3b on RBC leading to increased erythrophagocytosis and anaemia.

## Conflict of interest

The authors declare that they have no competing interests.

## Authors' contributions

MF and PED designed and managed the Mother Offspring Malaria Studies Project. MG designed and performed the study, analysed the results, and together with PED prepared the manuscript. GD supervised the laboratory work. All authors reviewed and approved the manuscript.
